# The Contribution of Density Functional Theory to the Atomistic Knowledge of Electrochromic Processes

**DOI:** 10.3390/molecules26195793

**Published:** 2021-09-24

**Authors:** Bruna Clara De Simone, Marta Erminia Alberto, Tiziana Marino, Nino Russo, Marirosa Toscano

**Affiliations:** Dipartimento di Chimica e Tecnologie Chimiche, Università della Calabria, 87036 Rende, CS, Italy; desimone@unical.it (B.C.D.S.); malberto@unical.it (M.E.A.); tmarino@unical.it (T.M.); m.toscano@unical.it (M.T.)

**Keywords:** DFT, TDDFT, electrochromic systems, redox potentials, excitation energies

## Abstract

In this review, we provide a brief overview of the contribution that computational studies can offer to the elucidation of the electronic mechanisms responsible for the electrochromism phenomenon, through the use of the density functional theory (DFT) and its time-dependent formulation (TDDFT). Although computational studies on electrochromic systems are not as numerous as those for other physico-chemical processes, we will show their reliability and ability to predict structures, excitation energies, and redox potentials. The results confirm that these methods not only help in the interpretation of experimental data but can also be used for the rational design of molecules with interesting electrochromic properties to be initiated for synthesis and experimental characterization.

## 1. Introduction

**Scheme 1:**
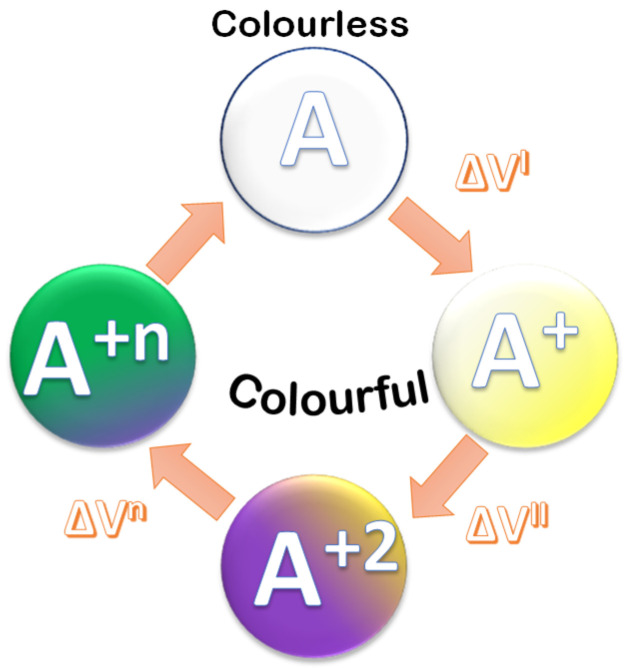
Schematic representation of a generic electrochromic process.

Electrochromism refers to the phenomenon in which a chemical system changes color following the application of an electric field of suitable value, returning to the original color once the applied field is eliminated. The change in the absorption band is due to the triggering of a redox reaction induced by the electric field. Compounds possessing more than two redox states are subject to different color changes as the applied electric field reaches the given redox potential. The whole process is described in Scheme 1.

Although the term electrochromic originally referred to a color change from a transparent (absorption in the UV region) to a colored species, today it is extended to all shifts of the absorption band in the entire electromagnetic spectrum caused by the application of an electric field.

Since Platt’s pioneering work in 1961 [1], electrochromism has been the subject of a large number of studies for its potential applications in different fields of modern technologies [2,3,4,5,6,7]. To date, electrochromic materials are mainly used for the manufacture of smart and intelligent windows that allow one to control the flow of heat and light [8,9,10], but show promising applications in glare reduction [11], mirroring [12], low-consumption display panels [13,14], transistors and diodes [15,16,17], catalysts [18], solar cells [19,20], camouflage materials [21], memory [22,23] and energy storage [24,25] devices, molecular machines [26], electrodes for batteries and supercapacitors [27,28,29,30,31], and antibacterial agents [32,33,34]. 

The most-studied systems are the viologen (1,1′-disubstituted-4,4′-bipyridinium salts) and its derivatives, for which many experimental studies have shown intrinsic properties that make them excellent candidates for a series of technological applications [35,36,37,38,39,40]. 

In recent years, however, the investigation has expanded to other promising organic and inorganic systems including those containing boron [41,42,43], quinodimethane derivatives [44,45], metal-phtalocyanine [46], metal oxides-based film [7,47,48], oligoaniline [49], antracenes [50,51], azacenes [52], polymethine and polyimides [53,54], and some organometallic complexes [55,56].

From a theoretical point of view, the studies are scarcer and have concerned some viologen-like [57], dithiolodithiole and thiophene [58], thiophene−pyrrole-containing rings [59], borepins [60], poly-dioxythiophene [61], and methyl ketone [62] systems. It is also worth mentioning certain investigations in which experimental data are coupled to theoretical data, demonstrating how the two approaches can be combined to provide useful insights on these interesting processes [36,39,53,56,63,64,65,66,67,68,69,70,71].

Despite this amount of work, accurate information at the atomistic level of electrochromic processes is somehow lacking, and modern theoretical methods can help to shed light on these aspects. Among these, those based on the Density Functional Theory (DFT) have proved to be very useful since they are able to provide accurate results even for medium to large systems. 

In this short review, we summarize the contribution of DFT methods in improving the current knowledge of the electrochromism phenomenon at the atomistic level. Particular emphasis will be devoted to those studies in which they computed and analyzed properties often difficult to obtain experimentally. Moreover, the outcomes of such investigations are not only useful to explain the material properties but also to build a rational design strategy to synthesize new molecules with given properties.

The computed properties we are interested in include structural determination, absorption behaviors and their origins, redox potentials, and electronic features.

**Scheme 2:**
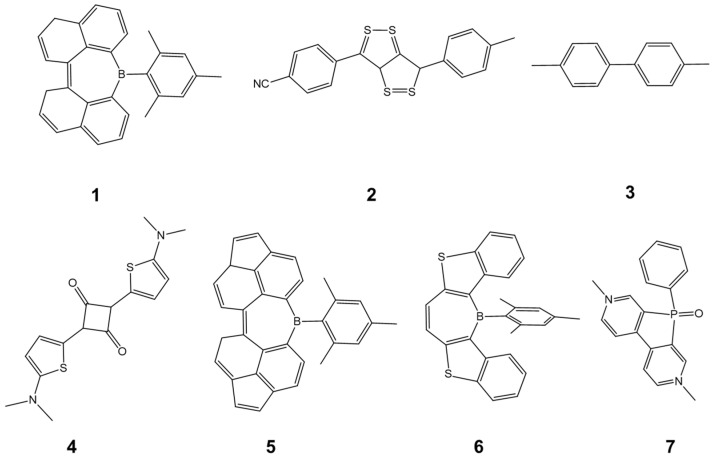
Structures of the considered systems. **1**: Quadruply benzannulated nonplanar borepin; **2**: 4-(6-(4-Metylphenyl)-[1,2]dithiolo[4,3-c][1,2]dithiol-3-yl)benzonitrile; **3**: Metylviologen; **4**: Squaraine; **5**: 5mesityl-5H-diacetonafto[5,6-bc:5′, 6′ef]borpin; **6**: dibenzo[b]thiophene-fused borepin; **7**: 3,7-diazabenzophosphole.

The structures of the considered systems are reported in Scheme 2.

## 2. Results and Discussion

### 2.1. Structural Determination

In order to show the accuracy of DFT methods in determining geometric parameters with accuracy, we report the calculated structures for quadruply benzannulated nonplanar borepin (1) [60] and for 4-(6-(4-Metylphenyl)-[1,2]dithiolo[4,3-c][1,2]dithiol-3-yl)benzonitrile (2) [58], using three different exchange and correlation functionals (see Scheme 2 and Table 1).

The values of the main geometrical parameters reported in Table 1 clearly show very good agreement with the previously reported X-ray crystallographic data [71].

In particular, for diphenyl- [1,2] dithiol [4,3-c] [1,2] we obtain C-C, C-S, and S-S bond length values that are very similar for the three functionals used and in the range of the experimental errors. The same agreement is found for the C-C-C, C-C-S, and C-S-S valence angles for which the error, with respect to the experimental counterpart [71], is about one degree.

Concerning the dihedral angles φ1 and φ2, a higher deviation from the experiment is noted for all the employed functionals. This can be explained by considering that, in the solid phase, the value of this parameter is influenced by the other molecules present in the crystalline structure, while our result is related to a single molecule.

For borepin molecules, we note that the maximum deviation from the experimental B-C and C-C bond lengths [41] is found in the case of the M06 functional, which does, however, provide values in the range of experimental error (Table 1).

Theoretical values obtained for the C-B-C valence angles and the C-C-C-C torsional angle obtained, using the three functionals, are in excellent agreement with the experimental X-ray results [41].

**Figure 1:**
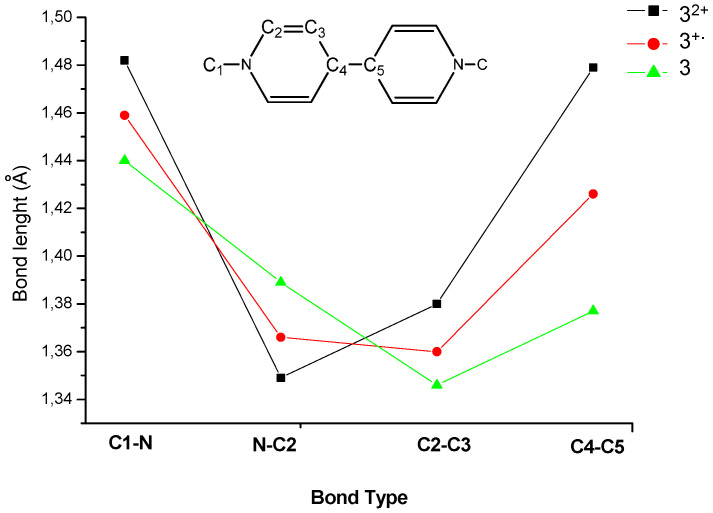
B3LYP/6–31+G* bond length variations during the oxidation process of **3**.

As previously mentioned, the experimental structures relative to the species with different oxidation states obtained upon the application of the electric field are often missing and difficult to measure. The use of calculations based on quantum mechanics can be useful to overcome this lack of information. In Figure 1, we report the main geometrical parameters obtained for viologen **3** as a function of the oxidation state.

The bond between the carbon atom of the CH_3_ group and the nitrogen of the pyridine ring (C1-N) undergoes a decrease, going from +2 to +1 radical to neutral charge states while the N-C2 increases following the consecutive addition of electrons. The C2-C3 and C4-C5 bond lengths involved in the conjugation of the ring decrease.

A more significant structural change is evidenced by analyzing the inter-ring dihedral angle that assumes a value of 39° in the case of the more-stable 32+ oxidation state but becomes planar in the case of 3+1 (0.13°) and 3 (0.02°) states.

These two examples account well for the importance of determining the structural variations induced by the change in the oxidation state on electrochromic systems, achievable by using reliable theoretical tools.

### 2.2. Excitation Energies

To assess the performance of DFT-based methods, we report the computed excitation energies for **3** [57] and squaraine **4** [70] in their different oxidation states by using the B3LYP, M06, and wB97XD exchange correlation functionals. The range-separated hybrid wB97XD functional has been chosen since it also takes into account weak interactions. The results are reported in Table 2 together with the corresponding experimental UV-Vis values [70,72].

For system **3**, the maximum deviation with respect to the experiment [72] (113 nm) is found in the case of the wB97XD functional that also shows an average error of about 57 nm considering all the oxidation states. The other two functionals used give similar results with average errors of 24 and 20 nm for M06 and B3LYP, respectively. The higher average error was obtained with the functional WB97XD, although it falls within an acceptable error range (0.1–0.3 eV), which could be due to the fact that at short range, it contains 22% of Hartree-Fock exchange and in the intermediate region is smoothly described by an error function parameter of 0.2.

In discussing the spectral behavior following the increase of the applied electric field, we note that they are in close agreement with the experiment. In fact, the dication **3**^2+^ species has a measured maximum absorption band of 337 that is well reproduced by all the employed functionals.

The addition of an electron generating the **3^+^** cation radical slightly red-shifts this band and two new bands in the Vis/NIR region appear (447 and 1100 nm). Both M06 and B3LYP functionals reproduce these spectral changes well. A further injection of an electron causes the **3** species to change the spectral feature that now presents two bands at 558 and 712 nm. Again, the obtained excitation energies for this oxidation state give results very close to the experimental counterparts. The analysis of the excitation behavior allows one to individuate the origin of the transitions. Table 2 shows the absorption at 368 nm (B3LYP) is mainly HOMO-LUMO (H→L) in nature (70 %), while it becomes βHOMO-1→LUMO in the radical cation. The other two peaks present in the Vis/NIR spectrum are due to the βH→L and αH→L transitions, respectively. Likewise, the computed excitation energies for the **3** neutral molecule allows one to assign the two recorded absorptions to the transitions HOMO→ LUMO + 3 and H→L, respectively (see Table 2).

The squaraines containing thiophene **4** are interesting since their structures contain donor–acceptor–donor moieties, which make them useful for a variety of applications. Molecule **4** is also important because it also has, in addition to the neutral one, positive (+1, +2) and negative (−1) oxidation states that are generated in Vis/NIR spectroelectrochemistry experiments. Table 2 shows how for these systems, the excitation energies obtained are also close to the experimental counterparts especially when the M06 and B3LYP functionals are used. A major deviation is found in the neutral species, but it is due to the possible intermolecular aggregation as demonstrated recently [70]. The average error obtained with these methods (about 25 nm) confirms their reliability in the treatment of electronic properties of complex systems. The analysis of the HOMO and LUMO orbitals involved in the transitions (Figure S1) allows one to establish that the band in the 450–500 nm range can be attributed to a charge-transfer between olate donors to the carbon atoms of the central cyclobutenediylium fragment and the thienyl moiety, for the **4**^2+^ species [70], while the main transition computed for molecule **4** is a π−π* band, as also demonstrated by the computed density difference plot with the first excited state considered (Figure S2).

### 2.3. Redox Potentials

The possibility to obtain reliable redox potentials at the theoretical level allows one to examine the energetics of the redox processes of a chemical species. Furthermore, it is useful to set up the spectroelectrochemical and cyclovoltametric experiments as we have indications on the potentials of the electric fields to apply.

Theoretically, the redox potentials can be obtained following the Nernst equation

E = −Δ(ΔGs)^redox^/nF (1)

where the Faraday constant (F) and the number of electrons transferred in the process (n) are present. The free energy change, Δ(ΔGs)^redox^, is computed on the basis of the Born–Haber thermodynamic cycle [73]. For a given system (A), the Δ(ΔGs)^redox^ can be obtained by computing the solvation energies (Δ Gs) for the neutral and cationic state by using the following equation:

Δ(ΔGsolv)^redox^ = ΔGg^redox^ + ΔGs(Red) − ΔGs(Ox) (2)

where ΔGg^redox^, ΔGs(Red), and ΔGs(Ox) are the change of standard Gibbs free energy for the gas phase reaction and solvation energies of reduced and oxidized species, respectively.

In order to compare the results with the experimental data, the computed redox potentials are provided relative to that of ferrocene (E(Fc+/Fc).

In Table 3, we report the computed reduction potentials (V) for a series of electrochromic materials obtained by using the M06 exchange correlation potential, that in our experience is the most suitable for these determinations [57,60]. Comparison with the relative experimental data [41,43,72,74,75] is, generally, quite good.

A more detailed comparison between computed and experimental data reveals that the difference varies from 0 to 0.43 and the average error turns out to be 0.15 and 0.7 V for the first and second reduction potentials, respectively.

### 2.4. Electronic Properties

**Figure 2:**
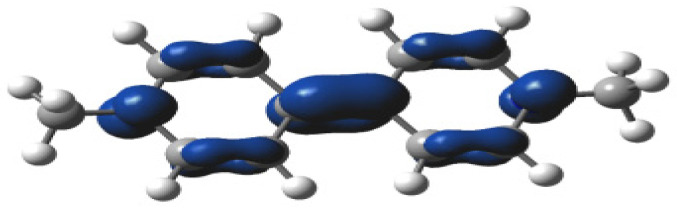
Electron spin distribution of **3**^+^ computed at the B3LYP/6–31+G* level of theory.

During the redox process, radical species can be obtained. As an example, in viologen-like systems, starting from the **3^2+^**, after the first reduction step, the **3^+^** radical species is formed. It is well known that the stability of the radicals strongly depends on their capability to delocalize the unpaired electron: Higher delocalization means great stability. Theoretical methods allow one to compute the spin distribution over the entire set of molecules as reported in Figure 2 for the **3^+^** radical.

The examination of this figure indicates that the unpaired electron is not only localized on the electron-rich nitrogen, but it is distributed on all the conjugated pyridine rings and on the C-C connecting bond with resulting system stabilization.

**Figure 3:**
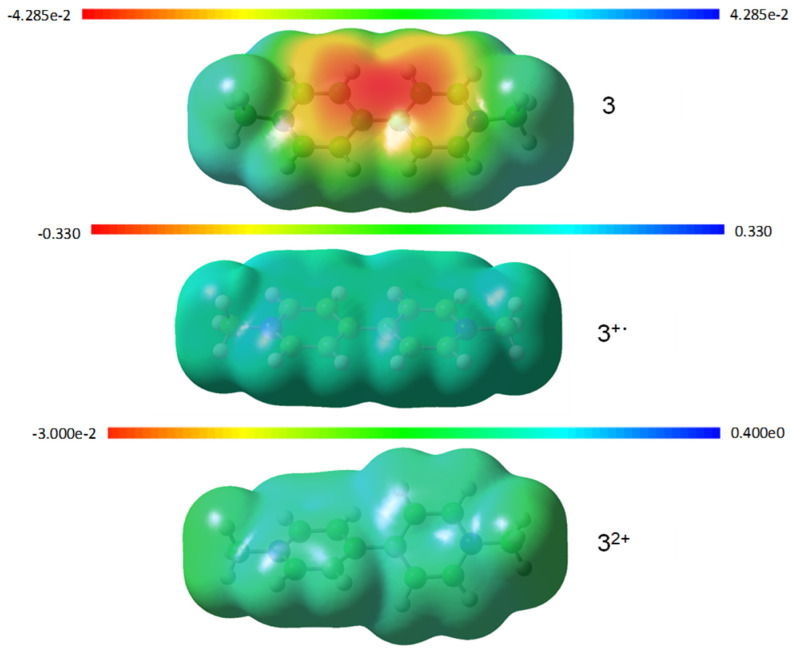
B3LYP/6–31+G* molecular electrostatic potential for the different oxidation states of **3** species.

Furthermore, the computation of the molecular electrostatic potential allows one to follow the charge flow during the reduction steps. The visualization of what happens after the electron addition for the **3**^2+^ + e→**3**^+^^.^ + e→**3** reduction behavior is given in Figure 3.

The analysis indicates how the positive charge, although reduced, continues to be distributed over the entire molecular surface upon the first reduction step. The second reduction process is characterized by different charge distribution with negative charges concentrated in the aromatic rings and between them.

**Figure 4:**
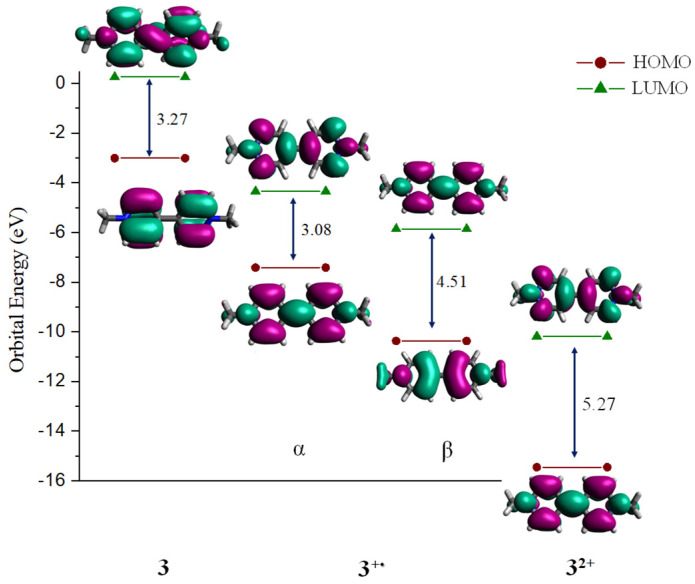
Frontiers molecular orbital values and orbital shape for the different oxidation states of **3**.

The knowledge of the frontier molecular orbitals and their composition can give further information on the electronic changes occurring during the redox processes. In Figure 4, we report the HOMO–LUMO energies and the orbital composition for the three oxidation states of **3**. It is interesting to note that both HOMO and LUMO orbitals are significantly destabilized in going from the +2 to 0 oxidation states. The HOMO–LUMO energy gap changes during the reduction process, assuming the highest value in **3**^2+^ (5.27 eV) and the lowest in the neutral (**3**) oxidation state. The changes in the molecular orbital composition evidence the effect of the addition of electrons in going from **3**^2+^ to **3,** especially for the HOMO orbital.

## 3. Materials and Methods

Full geometry optimizations in solvent media have been performed using the 6–31G* basis set in conjunction with the hybrid B3LYP [76,77], meta-hybrid M06 [78], and the range-separated hybrid wB97XD [79,80] exchange-correlation functionals. After the geometry optimization step, frequency calculations have been performed, at the same level of theory, to verify if the obtained structures are real minima. The unrestricted Kohn–Sham formalism has been used in the case of radical species. On these species, the analysis of the 〈S^2^〉 value (in all cases, reported at about 0.77) ensures the lack of spin contamination. The time-dependent density functional linear response theory (TDDFT) has been used to obtain the excitation energies starting from the previous optimization structures and employing the 6–31+G* basis set. Solvent effects have been taken into account by using the non-equilibrium polarizable continuum model [81]. All the computations have been performed with the Gaussian 09 code [82].

## 4. Conclusions

In this contribution, we have summarized the great potential of density functional theory in explaining the atomistic processes involved in the electrochromism of some important molecular materials. The main benefits of the theoretical computational investigation can be briefly summarized:

− A reliable prediction of the molecular structures with errors comparable to that coming from solid-state X-ray measurements. In addition, DFT computations allow one to determine the structures of systems with unstable oxidation states that are, consequently, difficult to measure experimentally in laboratory conditions;
− The reproduction of the excitation energies in all the molecular oxidation states is close to the experimental counterparts, with an error of about 0.2–0.3 eV. It is possible to assign the transitions and analyze the orbital origin and possible charge transfer processes;
− The redox potentials can be easily obtained with results very close to the experimental data;
− Spin charge distribution, molecular electrostatic potentials, charge distribution, and orbital picture substantially contribute to the interpretation of experimental data and shed light on their atomistic mechanisms.

We believe that the performances highlighted herein are sufficiently positive to encourage future theoretical studies with the aim of deepening knowledge on electrochromism in organometallic systems with multiple oxidation states, ionic materials, and molecules inserted in a polymeric or liquid crystal matrix, and at the same time, to rationally design new molecules with particular electrochromic and chemical properties to be suggested for syntheses and future applications.

## Figures and Tables

**Table 1:** (**a**) Main geometrical parameters for molecules 1 and 2, computed by using different exchange-correlation functionals by using the 6- 31+G* basis sets. The optimizations have been performed employing the Polarizable Continuum Model considering the chloroform and dichloromethane solvent for **1** and **2**, respectively. (**b**) Numeration of the considered parameters.

a)	X-Ray	B3LYP	M06	PBE0
**1**				
**B-C_1_**	1.563	1.564	1.559	1.560
**B-C_19_**	1.595	1.595	1.585	1.590
**C_9_-C_10_**	1.502	1.497	1.490	1.490
**C_1_BC_18_**	127.3	127.1	127.3	127.3
**C_1_BC_19_**	116.4	116.4	116.3	116.3
**C_8_C_9_C_10_C_11_**	38.9	39.2	39.2	39.0
**2**				
**C_1_-C_2_**	1.365	1.365	1.361	1.363
**C_2_-C_3_**	1.444	1.447	1.441	1.441
**C_2_-S_3_**	1.757	1.783	1.771	1.764
**S_3_-S_4_**	2.084	2.128	2.119	2.099
**C_1_-C_2_-S_3_**	126.5	125.7	126.0	126.3
**C_3_-C_2_-S_3_**	113.4	113.4	113.6	113.5
**C_2_-S_3_-S_4_**	95.8	95.1	95.1	95.4
**C_2_-C_3_-C_4_**	120.1	120.7	120.4	120.1
**φ1**	154.6	140.6	142.2	142.9
**φ2**	26.1	−34.7	−33.6	−32.7
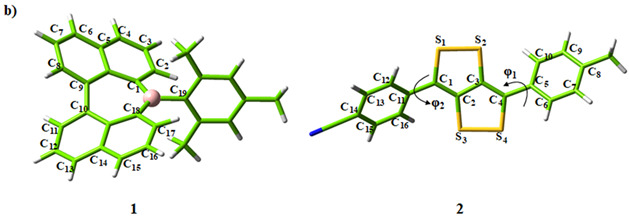				

**Table 2:** Excitation energies (nm) and oscillator strength of three and four molecules in different oxidation states computed in dichlorometane solvent by using different exchange-correlation potentials and the 6–31+G* basis set. The % of the transition is that obtained at the B3LYP level. Experimental data are taken from refs [70,72].

System	B3LYP	M06	wB97XD	Transition (%)	λ_exp_
**3** ^2+^	368 (0.23)	366 (0.24)	313 (0.30)	H→L (70)	337 [72]
**3** ^+^	399 (0.10) 433 (0.15) 1152 (0.11)	399 (0.13) 440 (0.12) 1169 (0.12)	355 (0.16) 403 (0.15) 1213 (0.15)	βH-1→L (84) βH→L (75) H→L (99)	400 [72], 447 [72] 1050–1100 [72]
**3**	553 (0.1) 721 (0.2)	557 (0.1) 698 (0.2)	455 (0.0) 599 (0.3)	H→L+3 (57) H→L (68)	558 [72], 712 [72]
**4** ^2+^	513 (1.39)	504 (1.49)	450 (1.74)	H→L (69)	450 [70]
**4** ^+^	575 (1.61) 842 (0.00)	581 (1.62) 825 (0.00)	565 (1.64) 733 (0.05)	βH→L (71) αH→L (74)	570 [70] 840 [70]
**4**	572 (1.67)	575 (1.64)	585 (1.70)	H→L (71)	660 [70]
**4** ^−^	494 (1.30) 709 (0.07)	487 (1.12) 690 (0.08)	444 (1.22) 643 (0.14)	αH→L (83) βH→L (84)	660 [70]

**Table 3:** Reduction potentials (V) for studied compounds computed by using the M06/6–31+G* level of theory. The used theoretical and experimental solvents are 5 and 6 in tetrahydrofuran; 3 and 7 in dichloromethane. ^a^ from refs [41,72,74,75].

Molecule	Potential	Theory (M06)	Experimental ^a^	Δ_theo-exp_
**5**	E^I^_red_	−1.60	−1.49	0.11
	E^II^_red_	−2.00	−1.84	0.16
**6**	E^I^_red_	−2.24	−2.14	0.10
**3**	E^I^_red_	−1.18	−1.09	0.09
	E^II^_red_	−1.42	−1.52	0.00
**7**	E^I^_red_	−0.74	−0.51	0.23
	E^II^_red_	−1.23	−1.00	0.23

## Data Availability

Not applicable.

## Supplementary Materials

The following are available online, Figure S1: Plots of the molecular orbitals involved in the transitions for **4** and **4**^2+^, Figure S2: Computed density difference plot for molecule **4** with the first excited state considered.
