# Supplementary material for: The Contribution of Density Functional Theory to the Atomistic Knowledge of Electrochromic Processes
Source: Molecules. 2021 Sep 24;26(19):5793. doi: 10.3390/molecules26195793 (PMC8510163; doi:10.3390/molecules26195793)
Supplement: Supplementary file 1 [file molecules-26-05793-s001.zip › molecules-1318185-supplementary.pdf]

Supporting Information

The contribution of density functional theory to the atomistic knowledge of electrochromic processes

Bruna Clara De Simone[1], **Marta Erminia Alberto**[1], **Tiziana Marino**[1], **Nino Russo**[1*], **Marirosa Toscano**[1]
Dipartimento di Chimica e Tecnologie Chimiche, Università della Calabria, Rende (CS), Italy
*Correspondence: nrusso@unical.it;

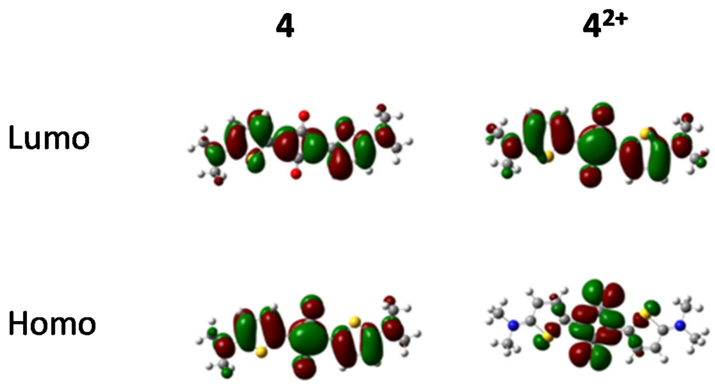

Figure S1. Plots of the molecular orbitals involved in the transitions for **4 and 4^{2+}**

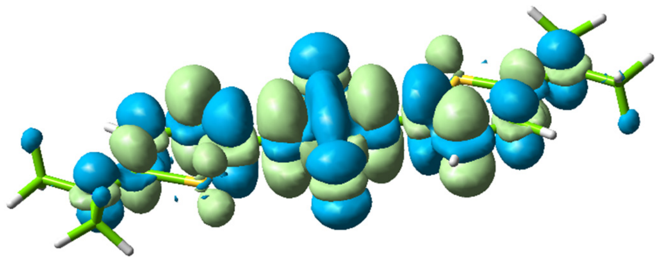

Figure S2. Computed density difference plot for molecule **4** with the first excited state considered (isodensity 0.000400 a.u.), the blue (green) regions indicating decrease (increase) in the electronic density upon electronic transition.

Table S1. Cartesian coordinates and total energies (in Hartree) for the optimized geometries (B3LYP/ 6- 31+G*)

1

Energy = -1143.84510063

**Atom**	**Coordinates (Angstroms)**		
	**X**	**Y**	**Z**
C	0.22349600	3.69967600	-0.99758400
C	-1.02447100	4.05543700	-0.54563000
C	-1.93559800	3.07580200	-0.07164800
C	-1.57372400	1.67946300	-0.11202000
C	-0.20621000	1.33604000	-0.42045000
C	0.63107200	2.35828200	-0.88107200
C	-3.19326500	3.48943100	0.43774000
C	-4.09273900	2.55631800	0.88881700
C	-3.80067700	1.18849100	0.73127000
C	-2.60603800	0.72321400	0.17919500
C	0.63809000	-2.35451600	0.88425400
C	-0.20212400	-1.33520600	0.42251000
C	-1.56831100	-1.68315300	0.11328200
C	-1.92563500	-3.08066700	0.07316500
C	-1.01179800	-4.05711700	0.54850200
C	0.23464200	-3.69710700	1.00133100
C	-2.60346900	-0.73035000	-0.17913000
C	-3.79603400	-1.19966300	-0.73225200
C	-4.08347100	-2.56849000	-0.88968200
C	-3.18146100	-3.49853300	-0.43732100
B	0.49202700	0.00141200	0.00075500
C	2.08752700	0.00306400	-0.00017800
C	2.81110500	-0.45432700	-1.12549400
C	4.21061400	-0.43852400	-1.11354700
C	4.93340700	0.00953800	-0.00426700
C	4.21171800	0.45952900	1.10513900
C	2.81231100	0.46619500	1.12218500
C	2.08685600	0.97492300	2.35158200
C	6.44470000	-0.01564000	0.00537500
C	2.08450700	-0.95833300	-2.35616200
H	0.91280800	4.44891300	-1.37689000
H	-1.33733100	5.09718500	-0.53810000
H	1.66152300	2.11567800	-1.11799300
H	-3.41704100	4.55256000	0.47865900
H	-5.04058100	2.85782700	1.32573400
H	-4.54847500	0.46325100	1.03421800
H	1.66760500	-2.10846100	1.12168600
H	-1.32130500	-5.09986900	0.54126400
H	0.92599100	-4.44394800	1.38166200
H	-4.54591300	-0.47694300	-1.03608100
H	-5.02987200	-2.87322400	-1.32748400
H	-3.40177500	-4.56239200	-0.47806800
H	4.74936700	-0.78690800	-1.99358900
H	4.75141200	0.81938900	1.98002400
H	2.79064500	1.24350200	3.14606200
H	1.39881600	0.22207100	2.75675700
H	1.48518500	1.86375700	2.12395700
H	6.85415000	0.09715600	-1.00441800
H	6.82673900	-0.96487300	0.40537800
H	6.85561100	0.78563500	0.62935800

H	1.39630900	-0.20414500	-2.75857200
H	1.48280900	-1.84772100	-2.13073200
H	2.78760200	-1.22486100	-3.15194500

2

Energy = -2340.02642946

Atom	Coordinates (Angstroms)		
	**X**	**Y**	**Z**
S	6.87780500	4.53508700	5.30567200
S	4.75721900	4.48588600	5.08176100
C	7.21229400	3.75738900	3.73288100
C	8.43155300	3.30895500	3.32529400
C	9.70982900	3.37842600	4.04795600
C	10.05926500	4.52186500	4.78855100
H	9.40771500	5.39079900	4.78686300
C	11.26452900	4.57823300	5.48623100
H	11.50979000	5.47718600	6.04616800
C	12.17001400	3.50978700	5.46344300
C	11.82790100	2.37849200	4.70616400
H	12.51309200	1.53527400	4.66493700
C	10.62503000	2.30837900	4.01150000
H	10.38240700	1.40837900	3.45366500
S	6.38088700	2.74496500	1.36431700
S	8.46987200	2.53940000	1.71533600
C	6.03077800	3.51850800	2.93199900
C	4.78821400	3.76239200	3.44133700
C	3.48811400	3.47356200	2.83071300
C	2.38344000	3.09550100	3.62401100
H	2.49602400	3.00410600	4.69963400
C	1.15564600	2.80172400	3.04941500
H	0.32117400	2.50003700	3.67349800
C	0.99367600	2.88463000	1.65452900
C	2.07830800	3.27590600	0.85186700
H	1.95425900	3.36148100	-0.22241500
C	3.30291200	3.57282700	1.43614200
H	4.10840500	3.92857500	0.80239100
C	13.46379200	3.56214300	6.23908600
H	13.37287700	3.02392400	7.19185200
H	14.28307000	3.09478500	5.68226100
H	13.75014100	4.59261500	6.47088600
C	-0.27113500	2.58044700	1.05719300
N	-1.29977600	2.33257800	0.57148900

3

Energy = -575.15731303

Atom	Coordinates (Angstroms)		
	**X**	**Y**	**Z**
C	2.85611900	1.18649000	-0.05666200
C	1.50512400	1.21298700	-0.04915500
C	0.69235800	-0.00001800	-0.04797200
C	1.50517500	-1.21299400	-0.04970100
C	2.85616600	-1.18645600	-0.05728300
H	3.45282800	2.09303200	-0.05849400

H	1.03242500	2.18864700	-0.05506900
H	1.03249000	-2.18865400	-0.05641100
H	3.45290800	-2.09297800	-0.05960900
C	-0.69228400	-0.00004000	-0.04780200
C	-1.50508800	-1.21302600	-0.04879000
C	-1.50506800	1.21295800	-0.04934500
C	-2.85609500	-1.18647900	-0.05604000
H	-1.03243300	-2.18870200	-0.05468200
C	-2.85607000	1.18646200	-0.05667500
H	-1.03235700	2.18860500	-0.05603500
H	-3.45283400	-2.09299900	-0.05767700
H	-3.45280700	2.09298200	-0.05879800
N	-3.58663800	-0.00002500	-0.09656900
N	3.58677600	0.00003800	-0.09745900
C	4.99488700	0.00000800	0.24833300
H	5.16923800	-0.00015100	1.33650400
H	5.47701400	-0.88561700	-0.17757300
H	5.47700100	0.88575900	-0.17732500
C	-4.99519900	0.00006200	0.24721000
H	-5.17110000	0.00018700	1.33513100
H	-5.47670200	0.88572400	-0.17932300
H	-5.47678500	-0.88564300	-0.17914800

3+
Energy = -575.04333852

**Atom**	**Coordinates (Angstroms)**		
	**X**	**Y**	**Z**
C	2.85744200	1.17837300	-0.01227600
C	1.49198500	1.20406200	-0.01177300
C	0.71605100	0.00000100	-0.01062100
C	1.49193200	-1.20408600	-0.01197200
C	2.85739800	-1.17845500	-0.01247100
H	3.45281700	2.08353700	-0.01192800
H	1.02073800	2.17858000	-0.01575200
H	1.02064700	-2.17858600	-0.01615000
H	3.45271400	-2.08365500	-0.01226500
C	-0.71605100	0.00002800	-0.01058900
C	-1.49196200	-1.20404600	-0.01171500
C	-1.49195500	1.20410300	-0.01191400
C	-2.85742000	-1.17838300	-0.01223400
H	-1.02069200	-2.17855400	-0.01565000
C	-2.85742000	1.17844600	-0.01242700
H	-1.02069200	2.17861200	-0.01604800
H	-3.45277600	-2.08355900	-0.01187700
H	-3.45275500	2.08363400	-0.01221800
N	-3.55798000	0.00003900	-0.01725300
N	3.55798200	-0.00006300	-0.01726700
C	5.02492800	0.00005300	0.05519600
H	5.35852600	0.00219300	1.09807200
H	5.41181000	-0.88818300	-0.44721700
H	5.41192600	0.88614600	-0.45094100
C	-5.02493000	-0.00006300	0.05512500
H	-5.35857900	-0.00156700	1.09798500

H	-5.41181600	0.88784700	-0.44786800
H	-5.41186900	-0.88648500	-0.45047500

32+
Energy = -574.87859060

Atom	Coordinates (Angstroms)		
	X	Y	Z
C	2.85427300	1.10622100	0.40198600
C	1.46903500	1.13033500	0.41005500
C	0.74448600	0.00114300	-0.00379800
C	1.47301300	-1.12590300	-0.41887800
C	2.85745300	-1.09549400	-0.41501800
H	3.45085900	1.95241100	0.72274300
H	0.97369100	2.02785500	0.76413000
H	0.98026100	-2.02358200	-0.77615400
H	3.45795600	-1.93877400	-0.73682000
C	-0.74448600	-0.00112100	-0.00380300
C	-1.46904500	-1.13031700	0.41005100
C	-1.47300400	1.12592100	-0.41888300
C	-2.85427400	-1.10619600	0.40197200
H	-0.97370300	-2.02783700	0.76412900
C	-2.85745300	1.09552200	-0.41502600
H	-0.98024900	2.02359800	-0.77616200
H	-3.45087900	-1.95237600	0.72272700
H	-3.45793500	1.93881300	-0.73682600
N	-3.52898700	-0.00713700	-0.00750000
N	3.52898700	0.00717100	-0.00748100
C	5.01974900	-0.00763600	0.02375700
H	5.34911600	-0.45740400	0.96385600
H	5.38560200	-0.58996000	-0.82224300
H	5.38617500	1.01576300	-0.05167700
C	-5.01974800	0.00753100	0.02377600
H	-5.34913300	0.45462400	0.96514400
H	-5.38562700	0.59225100	-0.82054700
H	-5.38613300	-1.01567300	-0.05458400

4
Energy = -1675.61483321

Atom	Coordinates (Angstroms)		
	X	Y	Z
C	0.01436700	0.92490900	0.88438200
C	1.33695800	0.42715300	0.45969000
C	1.56987100	1.66125500	-0.30620500
C	0.24727200	2.15900300	0.11847200
O	-0.90883000	0.50035300	1.58385800
O	2.49306300	2.08580600	-1.00569100
C	2.06478500	-0.73300700	0.69163400
C	1.70250900	-1.84483000	1.46186900
S	3.68405600	-0.97930900	-0.00717400
C	2.65672000	-2.85968300	1.50502500
H	0.74819900	-1.88138400	1.97583700
C	3.80466300	-2.56619400	0.74551000
H	2.53567400	-3.77792100	2.06433900

C	-0.48055700	3.31915700	-0.11349100
C	-0.11827400	4.43097300	-0.88373400
S	-2.09983300	3.56546800	0.58530400
C	-1.07248800	5.44582200	-0.92691500
H	0.83604200	4.46752200	-1.39769000
C	-2.22044000	5.15233600	-0.16741200
H	-0.95144000	6.36405600	-1.48623700
N	4.89115300	-3.34812100	0.56309100
N	-3.30694000	5.93425500	0.01497300
C	-3.41339400	7.18174200	-0.74003900
H	-3.54840600	6.99539200	-1.81350400
H	-4.27003600	7.74509400	-0.36927900
H	-2.51423400	7.78845000	-0.59726200
C	-4.48286900	5.41935400	0.70950800
H	-5.14964000	6.24960600	0.94480600
H	-5.02889300	4.68626300	0.10020900
H	-4.18708800	4.94326400	1.65025100
C	6.06709700	-2.83320500	-0.13140900
H	6.61317000	-2.10020700	0.47795900
H	6.73382000	-3.66346800	-0.36680800
H	5.77132300	-2.35700200	-1.07209600
C	4.99762600	-4.59560700	1.31810300
H	5.85421700	-5.15899100	0.94727200
H	5.13273700	-4.40925600	2.39155600
H	4.09843400	-5.20228200	1.17540400

4-
Energy = -1675.71483628

**Atom**	**Coordinates (Angstroms)**		
	**X**	**Y**	**Z**
C	0.07889200	1.00024100	0.98442000
C	1.33247500	0.41552100	0.46756300
C	1.50510200	1.58564500	-0.40668800
C	0.25153600	2.17038300	0.11019800
O	-0.77866000	0.65978200	1.81320900
O	2.36267800	1.92612200	-1.23544700
C	2.05335700	-0.76408100	0.70225100
C	1.77934800	-1.82280900	1.56345400
S	3.59193600	-1.07925400	-0.15216100
C	2.75573600	-2.85253400	1.56256000
H	0.89320000	-1.82871000	2.18885100
C	3.80477200	-2.62561900	0.69136900
H	2.68677000	-3.73148000	2.19252000
C	-0.46927000	3.35005500	-0.12436800
C	-0.19522900	4.40882500	-0.98551100
S	-2.00778300	3.66527400	0.73014800
C	-1.17153700	5.43862600	-0.98447800
H	0.69088800	4.41471000	-1.61095100
C	-2.22054400	5.21172300	-0.11325000
H	-1.10253600	6.31761900	-1.61436900
N	4.86800700	-3.46249500	0.37328300
N	-3.28368400	6.04866500	0.20498400
C	-3.39428900	7.26203200	-0.59332600
H	-3.62572700	7.05883200	-1.65265900

H	-4.18830000	7.88749800	-0.17712000
H	-2.45577400	7.82240300	-0.54722400
C	-4.55824300	5.43335900	0.56425600
H	-5.22891000	6.20724500	0.94762600
H	-5.04739900	4.93350600	-0.28814400
H	-4.41118200	4.69569000	1.35799000
C	6.14248100	-2.84710600	0.01385000
H	6.63156600	-2.34695500	0.86611500
H	6.81325600	-3.62100100	-0.36931300
H	5.99531700	-2.10967200	-0.78008500
C	4.97877000	-4.67569100	1.17183400
H	5.77287300	-5.30113000	0.75576100
H	5.21016400	-4.47224600	2.23112900
H	4.04033500	-5.23620400	1.12582800

4+

Energy = -1675.44834474

**Atom**	**Coordinates (Angstroms)**		
	**X**	**Y**	**Z**
C	0.01435700	0.90710700	0.88318900
C	1.35635100	0.42724300	0.45828100
C	1.56959600	1.67917400	-0.30654200
C	0.22760600	2.15905900	0.11840700
O	-0.89688100	0.46830800	1.57214600
O	2.48082800	2.11798600	-0.99548700
C	2.09418300	-0.70677200	0.68405300
C	1.72014500	-1.83598100	1.46423500
S	3.71922700	-0.92997400	-0.00291200
C	2.66701400	-2.82669900	1.51391900
H	0.75756900	-1.87469300	1.96115800
C	3.84020400	-2.51478600	0.75615600
H	2.55022300	-3.75436200	2.05770600
C	-0.51015200	3.29319000	-0.10700700
C	-0.13606700	4.42268600	-0.88675600
S	-2.13516900	3.51624400	0.58012700
C	-1.08284000	5.41352700	-0.93594300
H	0.82649100	4.46153200	-1.38370500
C	-2.25597300	5.10135100	-0.17823500
H	-0.96602100	6.34143300	-1.47930900
N	4.90941800	-3.28987100	0.61959900
N	-3.32527100	5.87624800	-0.04106500
C	-3.40385100	7.15143600	-0.77394500
H	-3.31492100	6.97922900	-1.85001400
H	-4.37052600	7.60792300	-0.56864100
H	-2.61217800	7.82898800	-0.44214000
C	-4.49152000	5.46372200	0.74682400
H	-4.96018300	6.35125400	1.17322800
H	-5.21549400	4.94074700	0.11215500
H	-4.18452700	4.80932800	1.56482700
C	6.07626400	-2.87903500	-0.16821500
H	6.80434600	-2.36410600	0.46830500
H	6.53911600	-3.76663500	-0.60097500
H	5.77151700	-2.21792000	-0.98154400
C	4.98785100	-4.56465700	1.35320600
H	5.95442300	-5.02137500	1.14790200
H	4.89921700	-4.39181200	2.42919300

H	4.19601700	-5.24227000	1.02192700

42+

Energy = -1675.24422143

Atom	Coordinates (Angstroms)		
	X	Y	Z
C	-0.02436500	0.86946500	0.81257900
C	1.33959600	0.38874300	0.39996200
C	1.54814500	1.64815500	-0.38655000
C	0.18624900	2.13121000	0.03103100
O	-0.92832500	0.43750100	1.49334700
O	2.45141800	2.08002600	-1.06807200
C	2.08358600	-0.71264500	0.65469400
C	1.69792500	-1.82683500	1.49644700
S	3.70393200	-0.94971800	-0.02322600
C	2.63859300	-2.79244500	1.59458600
H	0.73522200	-1.84019200	1.99386500
C	3.83954400	-2.49375500	0.82852100
H	2.53003400	-3.68448000	2.19510300
C	-0.54510100	3.24812900	-0.19026100
C	-0.14168800	4.39160100	-0.98299200
S	-2.16027400	3.48293000	0.50096900
C	-1.05781500	5.38473700	-1.02124600
H	0.81761600	4.40767100	-1.48688200
C	-2.26036600	5.07508700	-0.26174200
H	-0.93065600	6.30524400	-1.57379400
N	4.91642100	-3.23203700	0.74319300
N	-3.31518500	5.83645400	-0.12146400
C	-3.40452700	7.20681300	-0.66062400
H	-4.09234400	7.20748100	-1.51024300
H	-3.79992700	7.84893700	0.12914600
H	-2.42980500	7.57623600	-0.96459800
C	-4.50353100	5.39446600	0.63168200
H	-4.38675700	5.66826700	1.68466900
H	-5.37606300	5.90050800	0.21857000
H	-4.63980300	4.31662800	0.53468800
C	6.09031400	-2.81466700	-0.04604500
H	6.97741900	-3.26524300	0.39857500
H	5.97698500	-3.16847800	-1.07534200
H	6.19816400	-1.72943800	-0.02973500
C	5.05456800	-4.54716500	1.39793300
H	5.47754600	-5.24032200	0.66826600
H	5.73932400	-4.44581800	2.24439900
H	4.09432100	-4.92628400	1.73469500

5

Energy = -1296.24791922

Atom	Coordinates (Angstroms)		
	X	Y	Z
C	-4.51895200	-0.33810100	-1.14841500

C	-5.23886100	-0.01692000	0.00413900
C	-4.51433800	0.30914700	1.15615600
C	-3.11636600	0.32078800	1.16891100
C	-2.39312100	-0.00955200	-0.00234000
C	-3.11799000	-0.34291300	-1.16791300
C	-2.39403600	-0.71130500	-2.44706500
C	-2.38618000	0.69167200	2.44371800
B	-0.79860500	-0.00449200	-0.00436400
C	-0.09160700	1.35257000	-0.32304300
C	-0.08161700	-1.35625600	0.31394700
C	1.30672600	1.65398700	-0.09786700
C	1.64768100	3.01692000	-0.07387400
C	0.76504600	4.09129000	-0.37801600
C	-0.53832900	3.78893100	-0.70632100
C	-0.93535000	2.43322800	-0.64004300
C	2.40671400	0.74094200	0.13519900
C	3.62234200	1.29432100	0.57392000
C	3.89736900	2.67738000	0.65833700
C	2.90906800	3.55865800	0.27917400
C	-0.91803100	-2.44478100	0.62348700
C	-0.51010800	-3.79731600	0.68845300
C	0.79780100	-4.08782300	0.36771500
C	1.67320300	-3.00532200	0.07151100
C	1.32048500	-1.64538700	0.09595900
C	2.94143800	-3.53565200	-0.27420700
C	3.92473900	-2.64530500	-0.64502400
C	3.63722600	-1.26482900	-0.56033000
C	2.41399500	-0.72254600	-0.12890300
C	-6.75035400	-0.02175500	0.01474600
C	1.57543600	-5.32384000	0.21650400
C	2.83902100	-4.99751200	-0.17477300
C	1.53119600	5.33416100	-0.22433200
C	2.79487500	5.01939000	0.17605100
H	-5.05970100	-0.59392400	-2.05831400
H	-5.05189100	0.56343300	2.06881100
H	-1.69018000	0.07236600	-2.75422700
H	-1.80965400	-1.63237900	-2.32643600
H	-3.09701000	-0.86947500	-3.27129300
H	-3.08552600	0.85857400	3.26929200
H	-1.79614700	1.60814300	2.31618400
H	-1.68636400	-0.09500300	2.75260400
H	-1.26650200	4.55529400	-0.96013600
H	-1.98511100	2.21827200	-0.80746100
H	4.41496600	0.61544100	0.86605800
H	4.87422400	3.00850400	1.00187400
H	-1.97048700	-2.23914100	0.78555500
H	-1.23332600	-4.57043700	0.93585300
H	4.90668900	-2.96744700	-0.98250700
H	4.42577000	-0.57876600	-0.84660300
H	-7.15576900	-0.26815700	-0.97190600
H	-7.15344200	0.95602000	0.30717100
H	-7.14471200	-0.75539400	0.72948500
H	1.19109400	-6.32270500	0.38680400
H	3.64619000	-5.69074500	-0.38237800
H	1.13957800	6.32938700	-0.39930300
H	3.59471100	5.71990100	0.38757600

Energy = -1862.81378333

Atom	Coordinates (Angstroms)		
	X	Y	Z
C	3.90286200	7.88863900	6.82717100
C	2.76706400	9.92010700	7.51500600
C	1.81801500	10.71409000	8.16580500
H	1.83725600	11.79479000	8.05971100
C	0.85503700	10.08864900	8.94603500
H	0.10558800	10.68176300	9.46236800
C	0.85529700	8.69021000	9.06217700
H	0.10045700	8.20184200	9.67218600
C	1.80331900	7.91045400	8.41158100
H	1.76155700	6.83977100	8.53023400
C	2.80550900	8.50230800	7.60602200
C	4.63420700	8.90207200	6.18165500
C	5.76219900	8.87263000	5.31826100
H	6.10714300	9.84378000	4.96999000
C	6.47470900	7.80527500	4.85894900
H	7.30278400	8.05152000	4.19793900
C	6.29522900	6.41992400	5.11867100
C	6.76763200	3.95870600	4.97663900
C	7.22523500	2.65909500	4.73921700
H	8.07752900	2.48491100	4.08906300
C	6.56581300	1.60279400	5.35287300
H	6.90073800	0.58305100	5.18548200
C	5.46825100	1.85987600	6.18853800
H	4.95362800	1.03245400	6.66910600
C	5.02141300	3.15520900	6.41758600
H	4.17460500	3.30252200	7.06823700
C	5.65997400	4.26430600	5.81305200
C	5.36828400	5.71134800	5.90428100
C	3.27697800	5.37688100	7.56618200
C	3.59445100	5.03858700	8.90144200
C	2.75932800	4.17705700	9.62185700
H	3.01659900	3.92995600	10.65076100
C	1.60328200	3.62739800	9.05663800
C	1.29518200	3.97069300	7.73572300
H	0.39695600	3.56110700	7.27608000
C	2.10921900	4.82944300	6.98810400
C	4.83317200	5.60419000	9.56418300
H	4.93123800	5.24752600	10.59450100
H	4.81023900	6.70102000	9.59140600
H	5.74470600	5.31762800	9.02428800
C	1.72557900	5.16837200	5.56278000
H	0.80080400	4.66174900	5.26871400
H	2.50879500	4.87093900	4.85395300
H	1.57054500	6.24709700	5.43282900
B	4.21328100	6.35858600	6.73647100
S	4.03998600	10.53523800	6.49703400
S	7.47506400	5.39671800	4.29334500
C	0.72914600	2.67464600	9.83941200
H	1.06689300	1.63586400	9.72305400
H	0.74800900	2.90137800	10.91093500
H	-0.31163400	2.71576700	9.50063900

7

Energy = -1222.22290033

Atom	Coordinates (Angstroms)		
	**X**	**Y**	**Z**
C	-2.96801700	-2.47517500	-0.70508100
C	-2.63052000	-0.43681200	0.50315200
C	-1.29028600	-0.57798100	0.39513500
C	-0.68851800	-1.72906000	-0.29247700
C	-1.63665100	-2.67695600	-0.83649100
H	-3.70409700	-3.16586500	-1.10130000
H	-3.09761200	0.39236900	1.02544400
H	-1.29750000	-3.56427700	-1.36280000
C	1.29021100	-0.57853500	0.39479400
C	2.63053100	-0.43797600	0.50251300
C	2.96683700	-2.47643100	-0.70588500
C	1.63534800	-2.67760900	-0.83700100
C	0.68777100	-1.72933700	-0.29268500
H	3.09811800	0.39098100	1.02471800
H	3.70251100	-3.16741600	-1.10234400
H	1.29568000	-3.56472900	-1.36331500
C	0.00030400	2.05432400	0.09571200
C	0.00284500	3.27649800	0.77878400
C	-0.00208000	2.04580100	-1.30707400
C	0.00305800	4.47836800	0.06704500
H	0.00448500	3.26740800	1.86484200
C	-0.00186100	3.24649600	-2.01547700
H	-0.00412300	1.10049000	-1.84406300
C	0.00071300	4.46422200	-1.32847200
H	0.00500700	5.42446600	0.60239200
H	-0.00372200	3.23442500	-3.10245900
H	0.00085800	5.39962600	-1.88243000
P	0.00028100	0.50776900	1.07946100
O	0.00072400	0.81426700	2.55244700
N	3.50492900	-1.35247600	-0.06407200
N	-3.50546100	-1.35091800	-0.06323100
C	-4.92263300	-1.31452000	0.25441000
H	-5.22264600	-0.28346800	0.45948600
H	-5.50411300	-1.67469200	-0.60057100
H	-5.17170100	-1.93117700	1.13082300
C	4.92218200	-1.31673800	0.25328000
H	5.50331200	-1.67723300	-0.60180200
H	5.22273200	-0.28581900	0.45823800
H	5.17113200	-1.93346900	1.12967400
